# Aging- and obesity-related peri-muscular adipose tissue accelerates muscle atrophy

**DOI:** 10.1371/journal.pone.0221366

**Published:** 2019-08-23

**Authors:** Shunshun Zhu, Zhe Tian, Daisuke Torigoe, Jiabin Zhao, Peiyu Xie, Taichi Sugizaki, Michio Sato, Haruki Horiguchi, Kazutoyo Terada, Tsuyoshi Kadomatsu, Keishi Miyata, Yuichi Oike

**Affiliations:** 1 Department of Molecular Genetics, Graduate School of Medical Sciences, Kumamoto University, Kumamoto, Japan; 2 Division of Laboratory Animal Science, Kumamoto University, Kumamoto, Japan; 3 Department of Emergency Surgery, The First Affiliated Hospital of Harbin Medical University, Harbin, China; 4 Department of Immunology, Allergy, and Vascular Biology, Graduate School of Medical Sciences, Kumamoto University, Kumamoto, Japan; 5 Center for Metabolic Regulation of Healthy Aging (CMHA), Kumamoto University, Kumamoto, Japan; 6 Division of Kumamoto Mouse Clinic, Institute of Resource Development and Analysis (IRDA), Kumamoto University, Kumamoto, Japan; University of Houston, UNITED STATES

## Abstract

Sarcopenia due to loss of skeletal muscle mass and strength leads to physical inactivity and decreased quality of life. The number of individuals with sarcopenia is rapidly increasing as the number of older people increases worldwide, making this condition a medical and social problem. Some patients with sarcopenia exhibit accumulation of peri-muscular adipose tissue (PMAT) as ectopic fat deposition surrounding atrophied muscle. However, an association of PMAT with muscle atrophy has not been demonstrated. Here, we show that PMAT is associated with muscle atrophy in aged mice and that atrophy severity increases in parallel with cumulative doses of PMAT. We observed severe muscle atrophy in two different obese model mice harboring significant PMAT relative to respective control non-obese mice. We also report that denervation-induced muscle atrophy was accelerated in non-obese young mice transplanted around skeletal muscle with obese adipose tissue relative to controls transplanted with non-obese adipose tissue. Notably, transplantation of obese adipose tissue into peri-muscular regions increased nuclear translocation of FoxO transcription factors and upregulated expression FoxO targets associated with proteolysis (*Atrogin1* and *MuRF1*) and cellular senescence (*p19* and *p21*) in muscle. Conversely, in obese mice, PMAT removal attenuated denervation-induced muscle atrophy and suppressed upregulation of genes related to proteolysis and cellular senescence in muscle. We conclude that PMAT accumulation accelerates age- and obesity-induced muscle atrophy by increasing proteolysis and cellular senescence in muscle.

## Introduction

Sarcopenia, which is characterized as loss of skeletal muscle mass and strength seen in older individuals, is drawing attention as a major cause of decreased quality of life (QOL) due to physical inactivity [1, 2]. The number of individuals with sarcopenia is rapidly increasing as numbers of elderly people increase worldwide [3, 4]. Some patients with sarcopenia are overweight and exhibit severe physical inactivity, a pathology termed sarcopenic obesity (SOB) [5, 6]. How obesity and/or aging induces muscle atrophy has not been fully defined.

In the pathology of obesity-associated diseases, ectopic lipid accumulation in non-adipose tissue, such as liver or the cardiovascular system, accelerates development of conditions such as non-alcoholic steatohepatitis and atherosclerotic cardiovascular disease [7–9], suggesting that lipid accumulation functions in disease progression. A recent paper reported that adipose tissue accumulated in the space surrounding skeletal muscles as peri-muscular adipose tissue (PMAT) in some elderly or obese patients (10). In that study, the ratio of adipose-to-muscle tissue in leg, as estimated by ultrasonographic echo intensity, was inversely associated with muscle thickness in aging individuals [10], suggesting in the aging or obese, ectopic fat accumulation is positively correlated with muscle atrophy and subsequent sarcopenia development.

Here, using aged and obese mice, we show that PMAT seen as accumulation of ectopic fat surrounding skeletal muscle increases in parallel with muscle atrophy due to reduced type II myofiber cell size. RT-PCR analyses revealed comparable patterns of expression of genes associated with secreted factors in aging and obese adipose tissue, suggesting a common effect of PMAT on muscle atrophy. When we asked how PMAT affects muscle atrophy in mice, we found that the degree of denervation-induced muscle atrophy in mice transplanted with obese adipose tissue was greater than that seen in mice that transplanted with non-obese adipose tissue. Notably, obese adipose tissue transplanted *in vivo* activated FoxO transcription factors and increased expression of genes associated with ubiquitin-mediated proteolysis and cellular senescence in muscle. Conversely, in obese mice, PMAT removal attenuated denervation-induced muscle atrophy and suppressed changes in expression of genes associated with proteolysis and senescence in muscle. Overall, this study demonstrates that PMAT deposition accelerates age- and obesity-induced muscle atrophy by activating FoxO factors and their targets, mediating proteolysis and senescence in muscle, and promoting development of sarcopenia.

## Materials and methods

### Animal studies

Wild-type male mice (C57BL/6) and *db/db* (with control *+m/+m* mice) male mice (CLEA Japan Inc. Tokyo, Japan) were used in the present study. All mice were maintained in a pathogen-free facility (at the Center for Animal Resources and Development, Kumamoto University, Kumamoto, Japan) under controlled environmental conditions with a 12:12 hour light:dark cycle at a stable temperature of 23°C and fed water and normal diet (ND) (CE-2; CLEA Japan Inc., Tokyo, Japan) *ad libitum*.

Young and aged mice were fed a ND and evaluated from 3–6 months and 18–22 months of age separately. Obese mice were established by feeding high-fat diets (HFD-32; CLEA Japan Inc., Tokyo, Japan) for 12 or 24 weeks starting at 8-weeks-old. Control mice were fed normal chow (ND) (CE-2; CLEA Japan Inc., Tokyo, Japan) for 12 or 24 weeks. Relevant to *db/db* mice, 8-week-old *db/db* male mice and corresponding *+m/+m* control mice were fed a ND. Animal experiments were approved by the Kumamoto University Ethics Review Committee.

### Mouse denervation model and adipose tissue-transplantation surgery

A mouse denervation-induced muscle atrophy model was established as reported previously [11]. Briefly, ten-week-old male C57BL/6 mice were anesthetized with pentobarbital by intraperitoneal injection, and sciatic nerve of the right lower limb was resected. For transplant studies using inguinal white adipose tissue (iWAT) from HFD- or ND-fed mice, an incision was made in the surface of the gastrocnemius muscle of the right leg and 20 mg of inguinal adipose tissue from either ND- or HFD-fed mice was transplanted into the space around gastrocnemius muscle. When we used iWAT from *db/db* mice as obese adipose tissue, we followed the identical protocol but transplanted 20 mg of inguinal adipose tissue from either *+m/+m* mice or *db/db* mice. Two weeks later, mice were sacrificed for analysis.

### Computed tomography (CT)

Mice were anesthetized by intraperitoneal injection of pentobarbital, and gastrocnemius muscle and surrounding fat were assessed by X-ray attenuation in computed tomography (CT) images (La Theta; Aloka Ltd., Tokyo, Japan).

### Indirect calorimetry

Mouse daily activities and levels of O_2_ consumption (VO_2_, L/min) and CO_2_ production (VCO_2_, L/min) were measured using an indirect calorimetry system (MK-5000RQ, Muromachi Kikai Co., Ltd., Tokyo, Japan). VO_2_ and VCO_2_ were used to calculate the EE (kcal/day) using Weir’s equation in which EE = [(VO_2_ × 3.941) + (VCO_2_ × 1.11)] × 1.44, as previously reported [12].

### C2C12 culture and treatment of myotubes

C2C12 cells were cultured as described elsewhere [11]. Briefly, C2C12 cells were grown in growth medium containing Dulbecco’s modified Eagle’s medium (DMEM; WAKO 044–29765, Tokyo, Japan) supplemented with 10% fetal bovine serum, 100 U/ml penicillin, and 100 μg/ml streptomycin in a 5% CO_2_ humidified atmosphere at 37°C. When cells reached 60–80% confluence, medium was replaced with differentiation medium composed of DMEM containing antibiotics and 2% horse serum, typically ~96 hours after seeding. The medium was changed every 48 hours. 24 hours later when we confirmed myocyte fusion, to evaluate the effects of PAI-1 on myotube atrophy, we added 100 nM dexamethasone (DEX; Sigma-Aldrich D1756-25MG, St. Louis, MO, USA) in vehicle with or without 5 μg/ml PAI-1 (ab93068, Abcam, Cambridge, UK) to myotube cultures, as reported elsewhere [13].

### Staining procedures

Gastrocnemius muscle samples were dissected and fixed in isopentane in liquid nitrogen, and sliced into 8μm sections. Hematoxylin and eosin staining (H&E; Wako, Osaka, Japan) was performed to evaluate the basic muscle morphology. The cross-sectional area was analyzed using BZ-X analyzer software (Keyence, Osaka, Japan).

To assess fat accumulation in gastrocnemius, Oil Red O (Sigma-Aldrich Co. LLC, USA) staining was performed according to our previously published protocol [14]. Stained samples on slide glasses were imaged using a microscope (model BZ-9000; Keyence, Osaka, Japan).

ATPase staining was performed as reported elsewhere [15]. Briefly, we first mixed 8 ml 0.1 M sodium barbital (Nakalai Tesque, Kyoto, Japan) and 8 ml 0.18 M CaCl_2_ (WAKO, Tokyo, Japan) with 24ml deionized water to a final pH of 10.3 (Mixture 1). We then mixed 8ml 0.1M sodium barbital, 4 ml 0.18 M CaCl_2_ and 100 mg ATP disodium salt (Kohjin Co., Ltd., Tokyo, Japan) with 24ml deionized water to a final pH of 9.4 (Mixture 2). Sample sections were shaken in Mixture 1 for 15 min and then transferred them to Mixture 2 and shaken 45 min more. Samples were then rinsed in 1% CaCl_2_ 3 times over 10 min and treated with a 2% CoCl_2_ (Nakalai Tesque, Kyoto, Japan) solution for 3 min with shaking. Samples were rinsed with 0.01 M sodium barbital 8 times and then with deionized water for 3 min. We then added a 1% v/v solution of ammonium sulfide (Kanto Chemical Co., Inc., Tokyo, Japan) (1 ml stock NH_4_SO_2_ + 99ml D.I.H_2_O) to the staining jar for 1 min. Finally, samples were rinsed, dehydrated and mounted with coverlips.

### Quantitative real-Time PCR

Total RNA was extracted using TRIzol reagent according our previous protocol [14]. Briefly, 1.5μg total RNA was used for first-strand synthesis with cDNA Reverse Transcriptase and random primers. DNase-treated RNA was reverse-transcribed using a Prime Script RT reagent Kit (Takara Bio Inc, Shiga, Japan). Relative transcript abundance was normalized to that of 18S rRNA levels in mouse. Primer set sequences are shown in S1 Table.

### Western blotting

Western blotting was performed as described elsewhere [15]. Briefly, 10 μg cytoplasmic or nuclear protein was separated on SDS-PAGE and transferred to PVDF membranes. Membranes with nuclear protein were incubated with anti-FoxO3a (#12829) or anti-FoxO1 (#2880). Membranes with cytoplasmic protein were incubated with anti-p-FoxO3a (#2599), anti-p-FoxO1 (Ser256) (#9461), anti-p-AKT (Ser473) (#9271), anti-p-AKT (Thr308) (#4056) or anti-AKT (#9272; Cell Signaling Technology, Danvers, MA, USA) antibodies at 4°C overnight diluted 1:1000 in Solution 1 (TOYOBO, Co., Ltd., Osaka, Japan). After TBST washes, membranes were incubated with HRP-conjugated sheep anti-rabbit IgG (GE Healthcare Life Science, Piscataway, NJ, USA) diluted 1:2000 in Solution 2 at room temperature for 60 min. Internal controls were mouse anti-Hsc70 (sc-7298; Santa Cruz Biotechnology, Santa Cruz, CA, USA) for cytoplasmic immunoblotting and mouse anti-Histone H3 (#4499) (Cell Signaling Technology, Danvers, MA, USA) for nuclear immunoblotting. Hsc70 and Histone H3 were used for normalization.

### Statistical analyses

Results are reported as mean ± standard error (SEM). Statistical differences between two groups were determined using the unpaired two-tailed Student’s *t*-test. Statistical significance was considered to be *p*< 0.05.

## Results

### PMAT accumulation increases with muscle atrophy in aged mice

**Fig 1:**
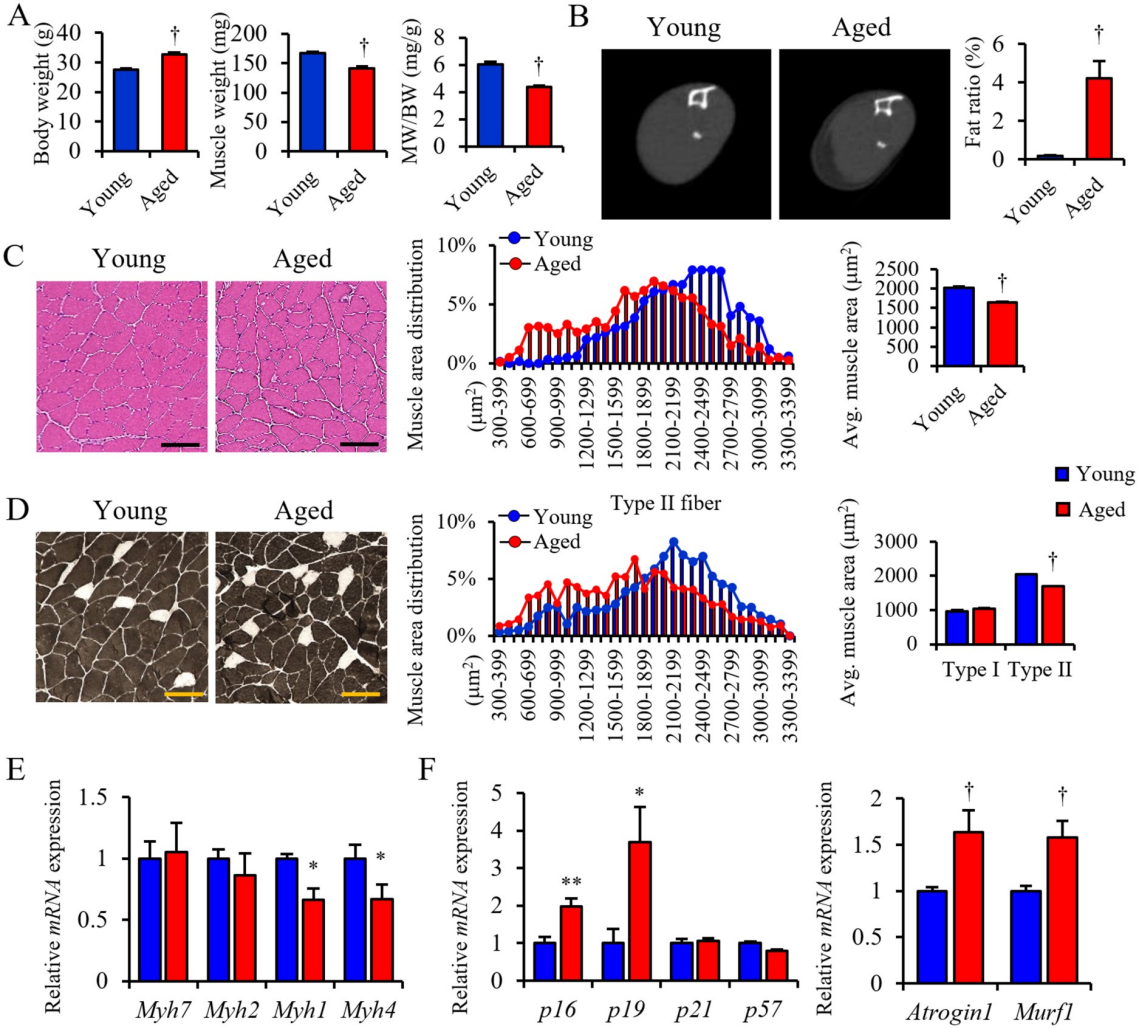
PMAT deposition is positively correlated with sarcopenia. (A) Body weight (BW), muscle weight (MW) and MW/BW ratio in young and aged mice. (B) Representative CT images (left panels) and the fat ratio based on CT analysis (right panel) in lower limbs of young and aged mice. (C) Representative HE staining (left panels), quantified distribution (middle panel) and average muscle fiber cross-sectional area (CSA) (right panel) in lower limbs of young and aged mice. (D) Representative ATPase staining (left panels), distribution CSA of type II muscle fibers (middle panel) and average CSA of type I (white cells in left panel) and type II (black cells in left panel) muscle fibers in lower limbs in young and aged mice (right panel) (n = 50–100, type I; n = 1200–1500, type II). (E, F) Relative levels of transcripts marking skeletal muscle myosin subtypes (*Myh7*, *Myh2*, *Myh1* and *Myh4*) (E), cellular senescence (*p16*, *p19*, *p21* and *p57*), and protein degradation (*Atrogin1* and *Murf1*) (F) in gastrocnemius of young and aged mice. Transcript levels were normalized to 18s mRNA; values in young mice were set to 1. Young mice were 3-6-months-old and aged mice were 18-22-months-old. (Scale bar: 100μm in c, d). (n = 6–8 per group in a, e, f). All data are presented as means±S.E. Statistical significance was determined by Student’s t-test. *, *p*<0.05; **, *p*<0.01; †, *p*<0.001.

To investigate whether muscle atrophy occurs in aged mice, we compared cellular and molecular characteristics of skeletal muscle in young (3–6 months-old) and aged (18–22 months-old) mice. First, body weight increased, but gastrocnemius muscle weight significantly decreased in aged relative to young mice (Fig 1A). Accordingly, computed tomography (CT) scan analysis of the lower leg revealed that skeletal muscle mass was less in aged relative to young mice and that aged mice showed significant increases of ectopic fat accumulation (PMAT) in the space surrounding atrophied hindlimb skeletal muscle, an effect not seen in young mice (Fig 1B). Histological analysis showed that the cross-sectional area (CSA) of muscle fiber of aged mice was smaller than that seen in young mice (Fig 1C).

To determine which type of muscle fiber underwent atrophy, we analyzed CSA of type I and type II muscle fibers separately and observed specific reduction in the CSA of type II but not type I muscle fibers of aged mice (Fig 1D). RT-PCR analysis confirmed that levels of mRNAs representing type II muscle fiber markers (*Myh1* and *Myh4*) but not the type I muscle fiber marker (*Myh7*) decreased in muscle of aged relative to young mice (Fig 1E). When we compared expression of genes associated with muscle atrophy in aged and young mice, genes associated with cellular senescence (*p16* and *p19*), protein degradation (*Atrogin1* and *Murf1*) (Fig 1F) and inflammation (*PAI-1*, *Il-1β* and *Tnfa*) were upregulated in aged muscle, whereas genes associated with differentiation and regeneration (*Myogenin*, *Myf5* and *MyoD*), mitochondrial lipid metabolism (*Pgc1α*, *Pparα/δ*, *Acadm*, *Acsl1*, *Cpt1α* and *Ucp3*), tissue fibrosis (*Tgf1β* and *collagen3a1*) and angiogenesis (*Cd31*) were downregulated in aged relative to young mice (S1A Fig). Immunostaining of muscle tissue with an antibody against 8-OHdG, a marker of DNA damage, revealed evidence of oxidative stress in gastrocnemius of aged mice, whereas minimal staining was seen in muscle of young mice (S1B Fig). We next asked whether muscle atrophy altered physical activity and energy expenditure in mice using metabolic chamber analyses. Expectedly, both physical activity and energy expenditure were significantly decreased in aged relative to young mice (S1C Fig), strongly suggesting that sarcopenia had developed in aged mice.

**Fig 2:**
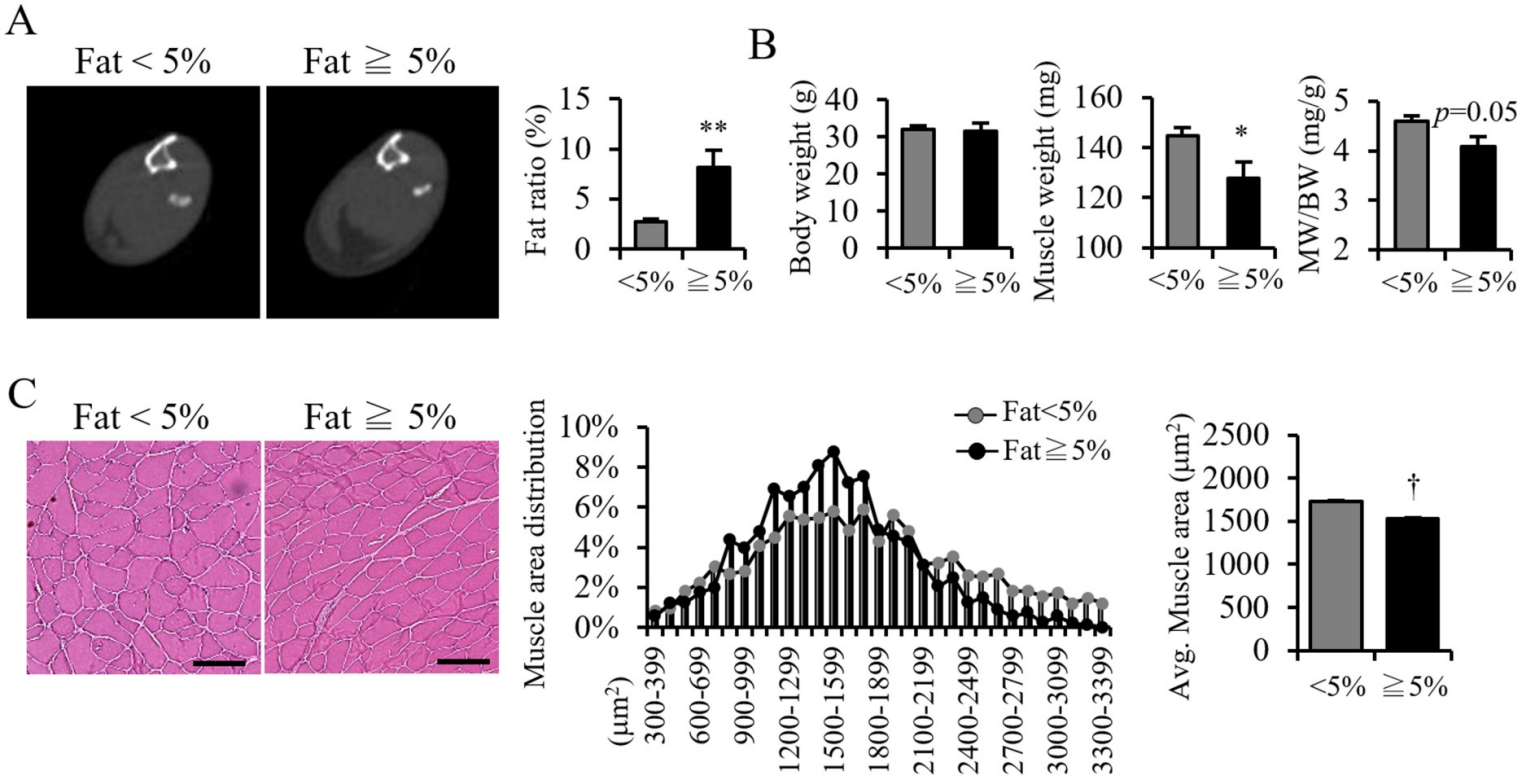
Aged mice exhibit PMAT increases with aggravated sarcopenia. (A) Representative CT images (left panels) and calculated fat ratio in lower limbs (right panel) in aged mice with less than (<5%) and equal to or more than 5% (≧5%) PMAT. (B) Body weight (BW), muscle weight (MW) and MW/BW ratio in aged mice in both groups (n = 6 per group). (C) Representative HE staining in aged mice in both groups (left panels; scale bar 100μm) and quantified distribution (middle panel) and average CSA of muscle fibers (right panel) (n = 1200–1500 per group) in aged mice of both groups. (Scale bar: 100μm in C). Data are presented as means±S.E. Statistical significance was determined by Student’s t-test. *, *p*<0.05; **, *p*<0.01; †, *p*<0.001.

Given that PMAT accumulation occurred only in aged mice (Fig 1B), we asked whether PMAT deposition might affect muscle atrophy. CT analysis revealed that the average of PMAT proportion in muscles of all the aged mice (32 in total) is 5%, Thus we chose 5% as a cut-off value and divided aged mice into two groups: one showing <5% PMAT and a another showing 5% PMAT or more (Fig 2A). Aged mice showing 5% or more PMAT also exhibited decreased muscle mass (Fig 2B) and muscular CSA (Fig 2C) compared to aged mice showing <5% PMAT, suggesting that the degree of PMAT deposition is associated with progression of muscle atrophy in aged mice. These observations further suggest that PMAT accumulation is a critical mechanism underlying acceleration of age-induced muscle atrophy.

### PMAT accumulation increases in obese mice exhibiting muscle atrophy

**Fig 3:**
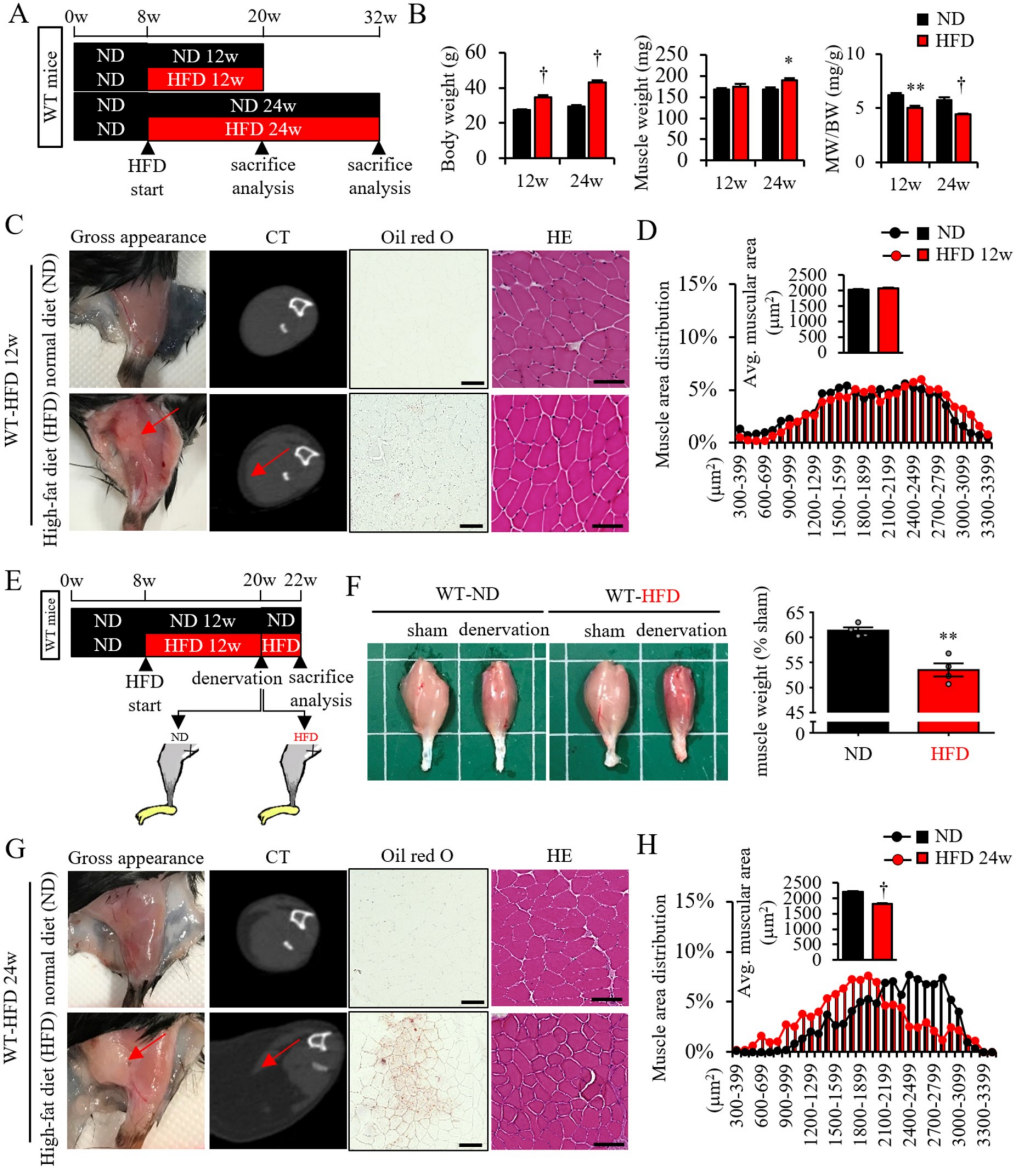
PMAT increases in obese mice and is accompanied by accelerated skeletal muscle atrophy. (A) Schematic showing protocol used in high fat diet (HFD) experiments. (B) Body weight (BW), muscle weight (MW) and MW/BW ratio in mice fed a HFD for 12 and 24 weeks relative to control mice fed a normal diet (ND) (n = 6 per group). (C and G) Representative gross appearance of lower limbs, CT images, Oil Red O staining and HE staining from WT mice fed a HFD (lower panels) for 12 (C) or 24 (G) weeks relative to mice fed a ND (upper panels). (D and H) Quantified distribution and average muscle fiber CSA (n = 1200–1500 per group) in WT mice fed indicated diets for 12 (D) or 24 (H) weeks. (E) Schematic showing sciatic denervation protocol with WT-ND and WT-HFD mice in which the latter group began a HFD at 8 weeks. (F) Representative samples of gastrocnemius (left panels) plus quantification of tissue weight relative to sham (shown as a %) (right panel) in mice fed a ND or HFD after 2 weeks of denervation or following sham operation. All data are presented as means±S.E. Statistical significance was determined by Student’s t-test. *, *p*<0.05; **, *p*<0.01; †, *p*<0.001.

Some patients with sarcopenia are overweight and characterized as having sarcopenic obesity (SOB) [5]. Moreover, ectopic lipid accumulation in non-adipose tissue, such as liver or cardiovascular tissue, accelerates development of non-alcoholic steatohepatitis and atherosclerotic cardiovascular disease [7, 8]. Thus, we asked whether ectopic fat accumulation around skeletal muscle tissue might accelerate progression of muscle atrophy in obese mice fed a high-fat diet (HFD) (Fig 3A). Compared to control mice fed a normal chow diet (ND) for 12 weeks, HFD feeding for 12 weeks increased body weight, decreased the muscle weight/body weight (MW/BW) ratio (Fig 3B), and promoted a small but significant accumulation of PMAT and intramuscular lipid (Fig 3C: PMAT was seen in visualizing and CT, intramuscular lipid was seen in Oil red O staining). Although muscular CSA was comparable in ND and HFD animals after 12 weeks (Fig 3C HE staining and Fig 3D), denervation-induced muscle atrophy was more severe in mice fed a HFD for 12 weeks relative to controls (Fig 3E and 3F). After 24 weeks, HFD feeding continued to accelerate body weight gain, decrease relative MW/BW (Fig 3B), and promote more accumulation of PMAT and intramuscular lipid relative to a ND diet, and mice in the HFD group showed a decreased muscle cell size (Fig 3G and 3H).

We then used *db/db* mice as a different obese mouse model and observed massive PMAT accumulation in a layer surrounding atrophied hindlimb skeletal muscle and significantly smaller cell size in skeletal muscle of the lower leg than that seen in control *+m/+m* mice (S2 Fig). Thus, these observations suggest overall that accumulated PMAT accelerates muscle atrophy in obese mice.

### Expression of secreted factors in PMAT resembles that seen in inguinal but not epididymal WAT

To determine whether PMAT accumulation directly accelerates muscle atrophy, we initially attempted to transplant PMAT from obese or non-obese young mice into the space surrounding skeletal muscle of non-obese young mice and assess whether denervation-induced muscle atrophy was accelerated. However, we could not conduct this analysis because young non-obese mice showed minimal PMAT. Thus, as a means of selecting an alternate transplant tissue, we assessed potential molecular differences between PMAT and subcutaneous white adipose tissue (WAT) or visceral WAT. Unlike PMAT, iWAT, which represents subcutaneous WAT, and epididymal WAT (eWAT), which represents visceral WAT, were abundant even in non-obese young mice. Therefore, we compared expression patterns of several presentative adipose tissue-related genes in PMAT versus iWAT and eWAT in aged mice. Expression of *Adiponectin*, *Leptin*, and *PAI*-1 was relatively high compared to that of *Il-6*, *Il-1β*, *Mcp-1*, *Il-10*, and *Tnf-α*, which was low, in PMAT, and these patterns were comparable in iWAT. eWAT, by contrast, showed high expression of *Il-6*, *Il-1β*, and *Mcp-1* relative to that of other adipocytokines, as shown in S3A Fig. Overall, these findings indicate that iWAT and PMAT exhibit similar expression patterns of mRNAs encoding adipose tissue-derived secreted factors.

### Patterns of iWAT-secreted factors in young versus aged mice correspond to those seen in ND versus HFD mice

When we assessed expression levels of genes encoding secreted factors in iWAT in young and aged mice, we observed increased expression of *Leptin*, *PAI-1*, and *Fgf-21* and decreased expression of *RBP4* in iWAT in aged relative to young mice (S3B Fig).

We next investigated expression levels of genes encoding secreted factors in iWAT in mice fed a ND versus a HFD. We observed relative increases in expression of *Leptin*, *PAI-1*, and *Fgf-21* and decreases in expression of *RBP4* and *Adipsin* in obese iWAT from mice fed a HFD (S3C Fig). Thus, patterns of gene expression associated with adipose tissue-derived secreted factors in iWAT from young versus aged mice were similar to those seen in ND versus HFD mice. Based on these results, we used iWAT rather than PMAT in the transplantation experiments reported below.

### Transplanted obese iWAT accelerates denervation-induced muscle atrophy in young non-obese mice

**Fig 4:**
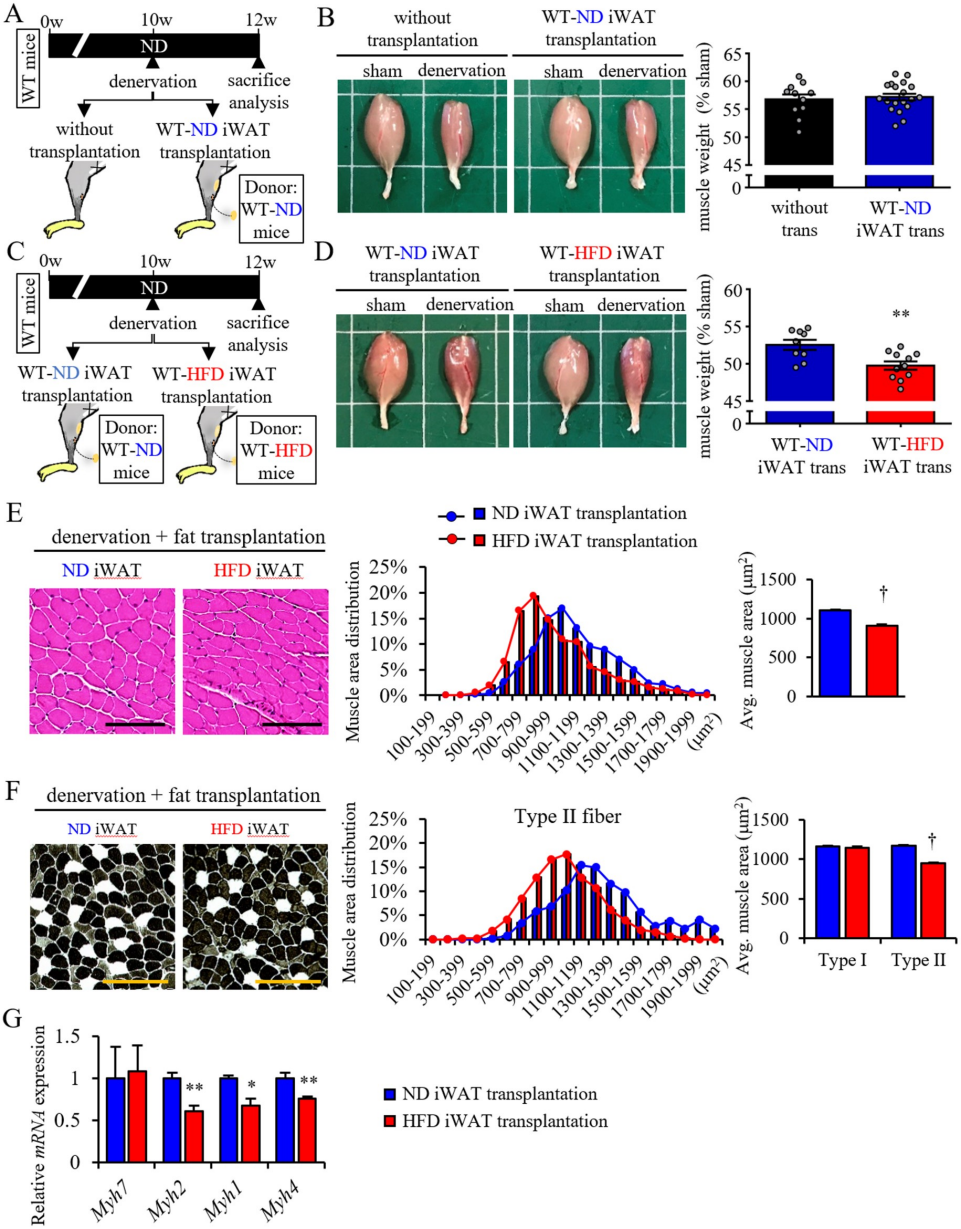
Transplant of adipose tissue to WT from obese mice aggravates muscle atrophy. (A and B) Effect of iWAT transplantation from WT mice fed a ND (WT-ND) on denervation-induced muscle atrophy. (A) Schematic showing sciatic denervation model with or without transplantation of iWAT from WT-ND. (B) Representative samples of gastrocnemius (left panels) plus quantification (right panel) of tissue weight relative to sham (shown as a %) in mice with or without iWAT transplantation after 2 weeks of denervation or following sham operation. (C-G) Effect of iWAT transplantation from WT mice fed a HFD (WT-HFD) on denervation-induced muscle atrophy compared to iWAT transplantation from WT-ND. (C) Schematic showing sciatic denervation model with or without transplantation of iWAT from WT-ND or WT-HFD. (D) Representative samples of gastrocnemius (left panels) plus quantification (right panel) of tissue weight relative to sham (shown as a %) in mice transplanted with ND or HFD iWAT after 2 weeks of denervation or sham operation. (E) Representative images of muscle HE staining (left panels), the calculated distribution (middle panel) and average muscle CSA (right panel) (n = 1200–1500 per group) in denervated mice transplanted with ND or HFD iWAT. (F) Representative images of muscle ATPase staining (left panels), the calculated distribution of type II (black cells in left panels) muscle fibers (middle panel), and average muscle CSA of type I and type II (white cells in left panels) muscle fibers (right panel) (n = 1200–1500 per group for type II muscle, n = 50–100 per group for type I muscle). (G) Relative levels of transcripts marking skeletal muscle myosin subtypes in muscle from mice transplanted with ND or HFD iWAT (n = 6 per group). Scale bar, 100μm in (E) and (F). All data are presented as means±S.E. Statistical significance was determined by Student’s t-test. *, *p*<0.05; **, *p*<0.01; †, *p*<0.001.

Initially, we found that transplantation of iWAT from non-obese young mice did not accelerate muscle atrophy (Fig 4A and 4B), while transplantation of iWAT from obese young mice did accelerate muscle atrophy (Fig 4C–4E). Moreover, histological analysis revealed that the CSA of type II, but not type I, muscle fibers specifically decreased in mice transplanted with obese iWAT relative to values seen in control mice transplanted with non-obese iWAT (Fig 4F). RT-PCR analysis indicated that transcript levels of *Myh1*, *Myh4*, and *Myh2* decreased in mice transplanted with obese iWAT compared to levels seen in mice transplanted with control non-obese iWAT, whereas *Myh7* expression was comparable in both (Fig 4G). These findings overall are comparable to phenotypes seen in atrophied muscle of aged mice (Fig 1).

We also observed accelerated denervation-induced muscle atrophy in mice transplanted by iWAT from *db/db* mice compared to mice transplanted with iWAT from *+m/+m* mice (S4A and S4B Fig). We also observed increased expression of *PAI-1* and *FGF21* and decreased expression of *RBP4* in iWAT from *db/db* mice (S4C Fig) as well as in iWAT from both obese mice fed HFD and aged mice (S3 Fig). Thus, transplanted obese iWAT accelerates denervation-induced muscle atrophy, even in young non-obese mice.

### Transplanted obese iWAT accelerates cellular senescence and FoxO-dependent proteolysis in muscle

**Fig 5:**
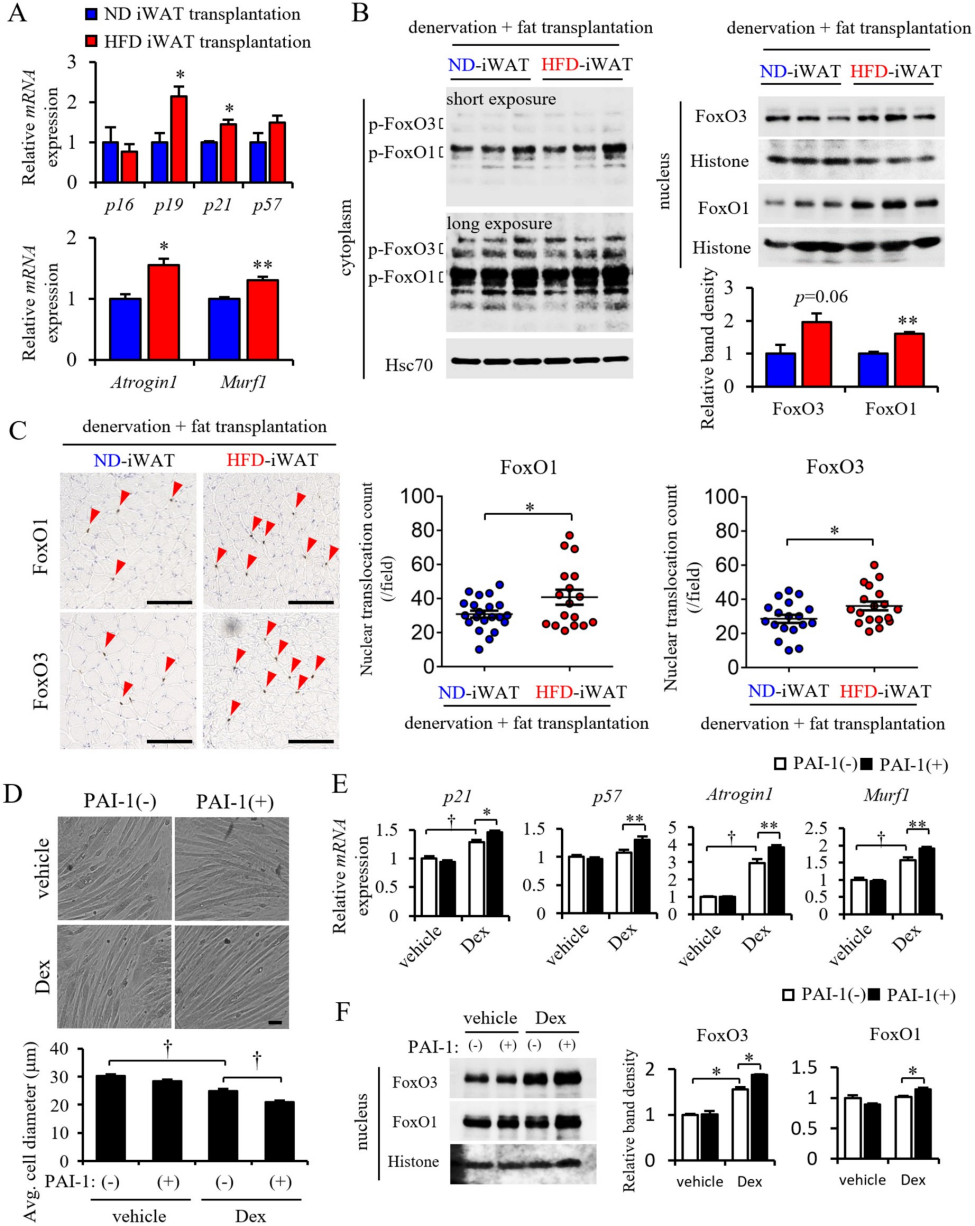
The presence of PMAT induces muscle senescence and up-regulates FoxO-Atrogin1/Murf1 signaling. (A) Relative transcript levels of senescence-related genes (*p16*, *p19*, *p21* and *p57*) and *Atrogin1 Murf1* in skeletal muscle of mice transplanted with ND or HFD iWAT (n = 6 per group). (B) Representative western blot images of phosphorylated FoxO3 and FoxO1 protein in the cytoplasm (left panel) and corresponding total protein in the nucleus (right top panel) of skeletal muscle from mice transplanted with ND or HFD iWAT. Corresponding quantification of total FoxO3 and FoxO1 protein in the nucleus (right bottom panel) (n = 3 per group). Hsc70 or Histone staining served as internal loading controls. (C) Representative images (left panels) and quantification (right panels) of the number of nuclear FoxO1- or FoxO3-positive cells in skeletal muscle from mice transplanted with ND or HFD iWAT. (D) Representative images (top panels) and quantified diameters (bottom panel) of differentiated C2C12 myotubes after PAI-1 and dexamethasone (Dex) treatment. (E) Relative expression of cellular senescence-related transcripts (*p21*, *p57*) and *Atrogin1* and *Murf1* mRNAs in differentiated C2C12 myotubes after PAI-1 and Dex treatment (n = 6–8 per group). (F) Representative western blot images (left panel) and corresponding quantification (right panels) of FoxO3 and FoxO1 proteins in the nucleus of differentiated C2C12 myotubes (n = 3 per group). Scale bar in (c) and (d), 100μm). All data are presented as means±S.E. Statistical significance was determined by Student’s t-test. *, *p*<0.05; **, *p*<0.01; †, *p*<0.001.

As shown in Fig 1 and also reported by others [16], expression of genes associated with cellular senescence, proteolysis, inflammation, stem cell/regeneration, energy metabolism, fibrosis, and angiogenesis is altered in atrophied muscle from aged mice [16]. Some previous reports have indicated that many of these factors are associated with sarcopenia development (for a review, see (3)). To assess which pathways are associated with PMAT-induced muscle atrophy, we undertook RT-PCR analysis, which revealed that expression of genes associated with cellular senescence, such as *p19* and *p21*, as well as protein degradation, such as *Atrogin1* and *Murf1*, increased significantly in atrophied muscles in mice transplanted with obese iWAT compared to non-obese iWAT (Fig 5A).

As *Atrogin1* and *Murf1* transcription is reportedly regulated by FoxO factors [17], we examined the intracellular localization of FoxO proteins in muscle by western blotting and immunohistochemical analyses. That analysis revealed significant increases in both FoxO1 and FoxO3 protein in the nuclear fraction from muscles of mice transplanted with obese iWAT compared to non-obese iWAT, while FoxO1 and FoxO3 protein levels in the cytoplasmic fraction were comparable between the two groups (Fig 5B). Immunohistochemical analysis revealed that the number of cells expressing nuclear FoxO1 and FoxO3 in muscle tissue of mice transplanted with obese iWAT significantly increased compared to the number seen in non-obese iWAT mice (Fig 5C).

Expression of genes encoding secreted proteins was similar in aged and obese adipose tissue (S3 Fig), suggesting that secreted proteins upregulated in both, such as PAI-1, act as common accelerators of muscle atrophy. Because local PAI-1 plays a critical role in muscle loss by inducing atrophy-related fibrosis [18], we asked whether PAI-1 treatment would accelerate changes associated with muscle atrophy using mouse C2C12 differentiated myotubes as a model. C2C12 cells were cultured and differentiated as described elsewhere [11]. After confirming myocyte fusion, we added dexamethasone (Dex) in vehicle with or without PAI-1 to myotube cultures. After 24 hours of Dex treatment, C2C12 myotube diameter was significantly reduced relative to the vehicle only group (Fig 5D). Interestingly, addition of PAI-1 accelerated reduction in myotube diameter seen following Dex treatment compared to Dex treatment only, while treatment with PAI-1 alone had no effect on myotube diameter (Fig 5D). PAI-1 treatment also accelerated Dex-dependent increases in expression of genes associated with cellular senescence (*p21*) and proteolysis (*Atrogin1*, *Murf1*) in myotubes and increased expression of *p57* (a marker of cellular senescence) in myotubes (Fig 5E). Furthermore, western blotting analysis revealed that PAI-1 treatment accelerated Dex-increased nuclear translocation of FoxO3 in myotubes as well as nuclear translocation of FoxO1 (Fig 5F). Taken together, we conclude that PMAT may accelerate obesity- and aging-related muscle atrophy by increasing secretion of factors such as PAI-1.

### PMAT removal attenuates denervation-induced muscle atrophy in obese mice

**Fig 6:**
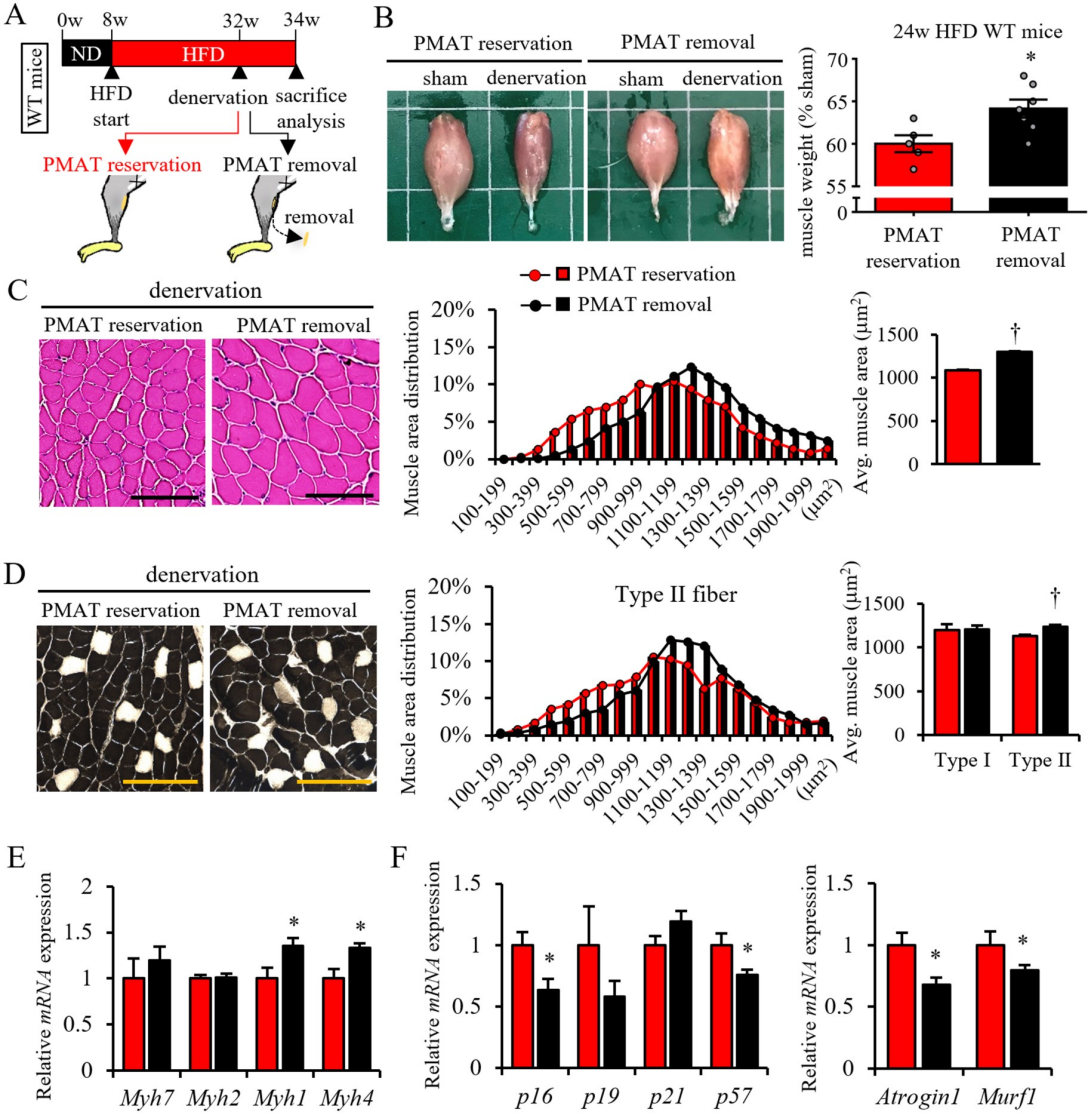
PMAT removal from obese mice protects against skeletal muscle atrophy. (A) Schematic showing protocol for sciatic denervation with or without PMAT resection in mice fed a HFD for 24 weeks. (B) Shown are representative samples of gastrocnemius (left panels) plus quantification (right panel) of tissue weight relative to sham (shown as a %) in mice with or without PMAT resection after 2 weeks of denervation or sham operation. (C) Representative images of muscle HE staining (left panels), calculated distribution (middle panel) and average muscle fiber CSA (right panel) (n = 1200–1500 per group) in the sciatic denervation model with or without PMAT resection. (D) Representative images of muscle ATPase staining (left panels), quantified distribution of type II muscle fibers (middle panel), and the average CSA of type I (black cells in left panels) or type II (white cells in left panels) muscle fibers (right panel) (n = 1200–1500 per group; scale bar, 100μm in c and d) from a sciatic denervation model, with or without PMAT resection. (E and F) Relative levels of transcripts marking skeletal muscle myosin subtypes (E), cellular senescence (F, left panel) and protein degradation (F, right panel) in the sciatic denervation model, with or without PMAT resection (n = 6 per group). All data are presented as means±S.E. Statistical significance was determined by Student’s t-test. *, *p*<0.05; †, *p*<0.001.

We next asked whether PMAT resection would attenuate denervation-induced muscle atrophy in obese mice fed a HFD (Fig 6A). To do so, we analyzed obese mice fed a HFD for 24 weeks, as these mice exhibit muscle atrophy with massive PMAT accumulation, as shown in Fig 3G. Based on macroscopic analysis, we observed less denervation-induced muscle atrophy in obese mice following PMAT removal than seen in mice without PMAT resection (Fig 6B). Histological analysis revealed that the CSA of muscle fibers, particularly type II fibers, in obese mice was larger following PMAT removal than it was without PMAT resection (Fig 6C and 6D). In agreement, we observed high expression of *Myh1* and *Myh4* mRNAs in muscle of obese mice following PMAT removal as compared to non-resected mice (Fig 6E). In addition, transcript levels of genes associated with cellular senescence, such as *p16*, and proteolysis, such as *Atrogin1* and *Murf1*, in muscles of obese mice following PMAT removal were significantly decreased compared to those seen in obese mice without PMAT resection (Fig 6F). These findings suggest overall that PMAT removal attenuates denervation-induced muscle atrophy of type II muscle fibers by blocking cellular senescence and proteolysis in those cells.

## Discussion

The present study reveals that muscle atrophy, which is characterized as predominant atrophic changes in type II muscle fibers, occurs in aged mice as is observed in elderly people. This condition is a sign of sarcopenia, and we observed that expression levels of genes associated with human sarcopenia development, including genes associated with cellular senescence, protein degradation, inflammation, regeneration, metabolism, fibrosis, and angiogenesis, also changed in atrophied leg muscle of aged mice, despite the fact that humans and mice walk on two and four legs, respectively. These findings suggest a common intrinsic mechanism underlying development of muscle atrophy in humans and mice. We also report ectopic fat accumulation layered around atrophied hindlimb skeletal muscle in aged and in obese mice, but not in young mice. Therefore, we focused on whether PMAT increases around skeletal muscle and how that accumulation affects muscle atrophy (S5 Fig).

Both aged and *db/db* mice presented significant PMAT accumulation with decreased muscle weight and CSA (Fig 1 and S2 Fig), indicating a potential association between PMAT and muscle atrophy. However, although mice fed a HFD for 24 weeks showed a decreased CSA relative to mice fed a ND for the same period, their muscle weight significantly increased (Fig 3B). On the other hand, HFD feeding for 12 weeks had no effect on CSA or muscle weight (Fig 3B), possibly because mice fed a HFD for 12 weeks showed only mild accumulation of intramuscular lipid (Fig 3C). By contrast, mice fed a HFD for 24 weeks showed a marked increase in intramuscular lipid and intermuscular adipose tissues (Fig 3G). These increases may greatly exceed the effects of muscle atrophy.

Interestingly, we observed similar patterns of expression of genes encoding secreted factors in aged and obese adipose tissues, suggesting that PMATs act as a common accelerator of obesity- and aging-related muscle atrophy via those secreted proteins, among them, PAI-1, Leptin, and Fgf21. Circulating PAI-1 levels increase in older and obese humans, suggesting that PAI-1 contributes to aging- and obesity-related disease [19–21]. A previous study reported no correlation between systemic levels of PAI-1 and sarcopenia [22]. However, unlike systemic PAI-1, local PAI-1 plays a critical role in muscle loss by inducing atrophy-related fibrosis [18], suggesting that PMAT-derived PAI-1 induces muscle atrophy in a paracrine manner. In the present study, we also showed that PAI-1 expression increases in muscles of aged mice, suggesting that this change in gene expression in both PMAT and muscle tissue may accelerate muscle atrophy.

Moreover, analysis performed in *in vitro* culture of C2C12 differentiated myotubes in the present study revealed that PAI-1 treatment increases expression of genes associated with cellular senescence and proteolysis, both critical for sarcopenia development. In contrast to PAI-1, FGF21 reportedly prevents muscle loss [23], suggesting that increased FGF21 might counteract muscle atrophy. On the other hand, leptin is thought to have little effect on muscle atrophy, as we observed muscle atrophy in leptin receptor-deficient *db/db* obese mice (S2 and S3 Figs). Thus, we conclude that PAI-1 is a strong candidate factor for accelerating muscle atrophy. However, adipose tissues secrete various bioactive substances (adipokines), such as pro- and anti-inflammatory cytokines, chemokines, and growth factors [24]. Here, we examined expression of a limited number adipokines, including PAI-1, in iWAT; therefore, we cannot exclude the possibility that other factors secreted from PMAT contribute to acceleration of muscle atrophy.

Some have reported that, except PMAT, obesity also induces intramuscular fat accumulation [25]. Interestingly, PMAT is three times greater than that of intramuscular fat in a tendon division model [26], results similar to ours in obese mice (Fig 3C and 3E, S2B Fig). These observations suggest that increases in intracellular lipid either parallel or are a consequence of PMAT development in obesity. That is why we focused here on the role of PMAT in muscle atrophy. However, other studies report that intramuscular lipids play a direct lipotoxicity role in muscle [27, 28], therefore, although the amount of intracellular fat is far less than the amount of PMAT, we can’t exclude the different lipid components, in aged and obesity mice compared to their young and lean mice, are also contributed to muscle atrophy. We think it would be of interest to investigate the role of lipotoxicity in inducing intracellular lipid in future analysis of muscle atrophy.

In our investigation of PMAT effects on aging- and/or obesity-related muscle atrophy, we excluded other influencers that might be associated with aging- and/or obesity-related systemic changes by performing two experiments: 1) transplantation of adipose tissue into the space of surrounding muscle of young non-obese mice, and 2) removal of PMAT from obese mice. We observed increases of FoxO transcription factors in the nucleus and subsequent increases in transcription of *Atrogin1* and *MuRF1* in skeletal muscle in denervation-induced muscle atrophy model mice, suggesting that PMAT accelerates muscle atrophy through by activating FoxO-dependent ubiquitin-mediated proteolysis in skeletal muscle. Both *Atrogin1* and *Murf1* mRNAs, which encode atrophy-related ubiquitin ligases, increase in aged skeletal muscle [29, 30], likely leading to increased proteolysis and sarcopenia development [31, 32]. Interestingly, PAI-1 deficiency reportedly suppresses *Atrogin*1 and *Murf*1 expression, while PAI-1 treatment increases *Atrogin*1 and *Murf*1 expression in C2C12 myocytes [13]. Based on these findings and our own, we conclude that increased PAI-1 derived from PMAT induces *Atrogin*1 and *Murf*1 transcription to accelerate proteolysis and subsequent muscle atrophy. Our study also suggests a role for enhanced cellular senescence in PMAT-induced muscle atrophy. In support of this idea, others have shown that PAI-1 inhibition reportedly antagonizes doxorubicin-induced upregulation of *p16* and *p21* mRNAs in endothelial cells, fibroblasts and cardiomyocytes [33], suggesting that PAI-1 accelerates cellular senescence. FoxO factors also reportedly increase transcription of *p16*, *p19* and *p21* [34–37], suggesting that PMAT-induced activation of FoxOs underlies upregulated senescence in muscle cells. Taken together, we conclude that aging and/or obese PMAT directly induces skeletal muscle atrophy by increasing muscle cellular senescence and proteolysis, and that PAI-1 secreted from PMAT is a strong candidate factor for accelerating muscle atrophy.

Various signaling pathways, such as p38 MAPK and JNK, reportedly activate FoxO in muscle cells [38]. Recently, it was reported that mTORC1 promotes denervation-induced muscle atrophy through activating FoxO [39]. However, it is unclear whether PAI-1 regulates mTORC1 signaling to accelerate FoxO nuclear accumulation. Further studies are required to define molecular mechanisms underlying PAI-1-mediated increases in FoxO nuclear accumulation in muscle.

In summary, aging and obesity lead to muscle atrophy and decreased muscle strength, which results in decreased quality of life due to physical inactivity. Our study reveals that PMAT accumulation accelerates age- and obesity-induced type II muscle fiber atrophy by increasing proteolysis and cellular senescence in muscle. Accordingly, in obese mice, denervation-induced muscle atrophy was attenuated by PMAT removal. Our work overall reveals an important mechanism underlying progression of skeletal muscle atrophy in older and/or obese subjects.

## Supporting information

S1 Fig Characteristics of skeletal muscles in aged mice compared to young mice. (A) Relative levels of transcripts marking inflammation (*Cd68*, *F4/80*, *PAI-1*, *Mcp-1*, *Il-6*, *Il-1β* and *Tnfα*), muscle cells differentiation and regeneration (*Myogenin*, *Myf5*, *MyoD* and *Myostatin*), mitochondrial lipid metabolism (*Pgc1α*, *Pparα*, *Pparδ*, *Acadl*, *Acadm*, *Acads*, *Acsl1*, *Cpt1α*, *Cpt1β* and *Ucp3*) and angiogenesis (*Tgf1β*, *collagen1*, *collagen3a1*, *Vegfα* and *Cd31)* in gastrocnemius of young and aged mice. Transcript levels were normalized to 18s mRNA; values in young mice were set to 1. (B) Representative images of 8-OHdG immunostaining to detect oxidative stress in gastrocnemius of young and aged mice. (C) Activity (left) and energy expenditure (EE) (right) in young and aged mice, as measured by indirect calorimetry. Young mice were 3-6-months-old and aged mice were 18-22-months-old. (Scale bar: 100μm in B). (n = 6–8 per group in A, C). All data are presented as means±S.E. Statistical significance was determined by Student’s t-test. *, *p*<0.05; **, *p*<0.01; †, *p*<0.001.
(TIF)

S2 Fig Diabetic mice exhibit skeletal muscle atrophy. (A) Body weight (BW) and muscle weight (MW) of *+m/+m* and *db/db* mice (n = 6 per group). (B) Gross appearance of lower limbs and representative images of CT, Oil Red O staining and HE staining from *+m/+m* and *db/db* mice. Scale bar, 100μm (C) Quantification of muscle CSA in *+m/+m* and *db/db* mice (n = 1200–1500 per group). Data are presented as means±S.E. Statistical significance was determined by Student’s t-test. †, *p*<0.001.
(TIF)

S3 Fig Analysis of mRNA expression in adipose tissue. (A) Relative expression of transcripts encoding adipokines (*Adiponectin* and *Leptin*) and pro-inflammatory cytokines (*PAI-1*, *Il-6*, *Il-1β*, *MCP-1*, *Il-10* and *Tnfα*) in eWAT, iWAT and PMAT from WT mice fed a HFD for 12 weeks (n = 2 per group). (B and C) Relative expression of genes associated with adipocytes or encoding pro-inflammatory cytokines in iWAT from young and aged mice (B), or from 12-week ND and HFD mice (C) (n = 6 per group). Transcript levels were normalized to 18s mRNA. Values in young iWAT or ND iWAT were set to 1. Data are presented as means±S.E. Statistical significance was determined by Student’s t-test. *, *p*<0.05; **, *p*<0.01; †, *p*<0.001.
(TIF)

S4 Fig Transplantation of adipose tissue from diabetic mice accelerates skeletal muscle atrophy. (A) Schematic illustrating the sciatic denervation model with iWAT transplantation from *+m/+m* or *db/db* mice. (B) Representative samples of gastrocnemius (left) plus quantification of tissue weight relative to sham (indicated as a %) in mice transplanted with iWAT from *+m/+m* or *db/db* mice after 2 weeks of denervation or sham operation. (C) Relative levels of mRNAs associated with adipocytes or encoding pro-inflammatory cytokines in iWAT of *+m/+m* or *db/db* mice (n = 6 per group). Transcript levels were normalized to 18s mRNA. Values in *+m/+m* mice were set to 1. All data are presented as means±S.E. Statistical significance was determined by Student’s t-test. *, *p*<0.05; **, *p*<0.01.
(TIF)

S5 Fig Model showing role of PMAT in skeletal muscle atrophy (sarcopenia). In aging or obesity, PMAT (in particular, PMAT secreting PAI-1) promotes nuclear translocation of FoxO transcription factors in skeletal muscle, accelerating skeletal muscle cell senescence and proteolysis and leading to atrophy (sarcopenia).
(TIF)

S1 Table Sequences of mouse primers used for quantitative RT-PCR. (DOCX)
