# Supplementary figures and images for: Aging- and obesity-related peri-muscular adipose tissue accelerates muscle atrophy
Source: PLoS One. 2019 Aug 23;14(8):e0221366. doi: 10.1371/journal.pone.0221366 (PMC6707561; doi:10.1371/journal.pone.0221366)

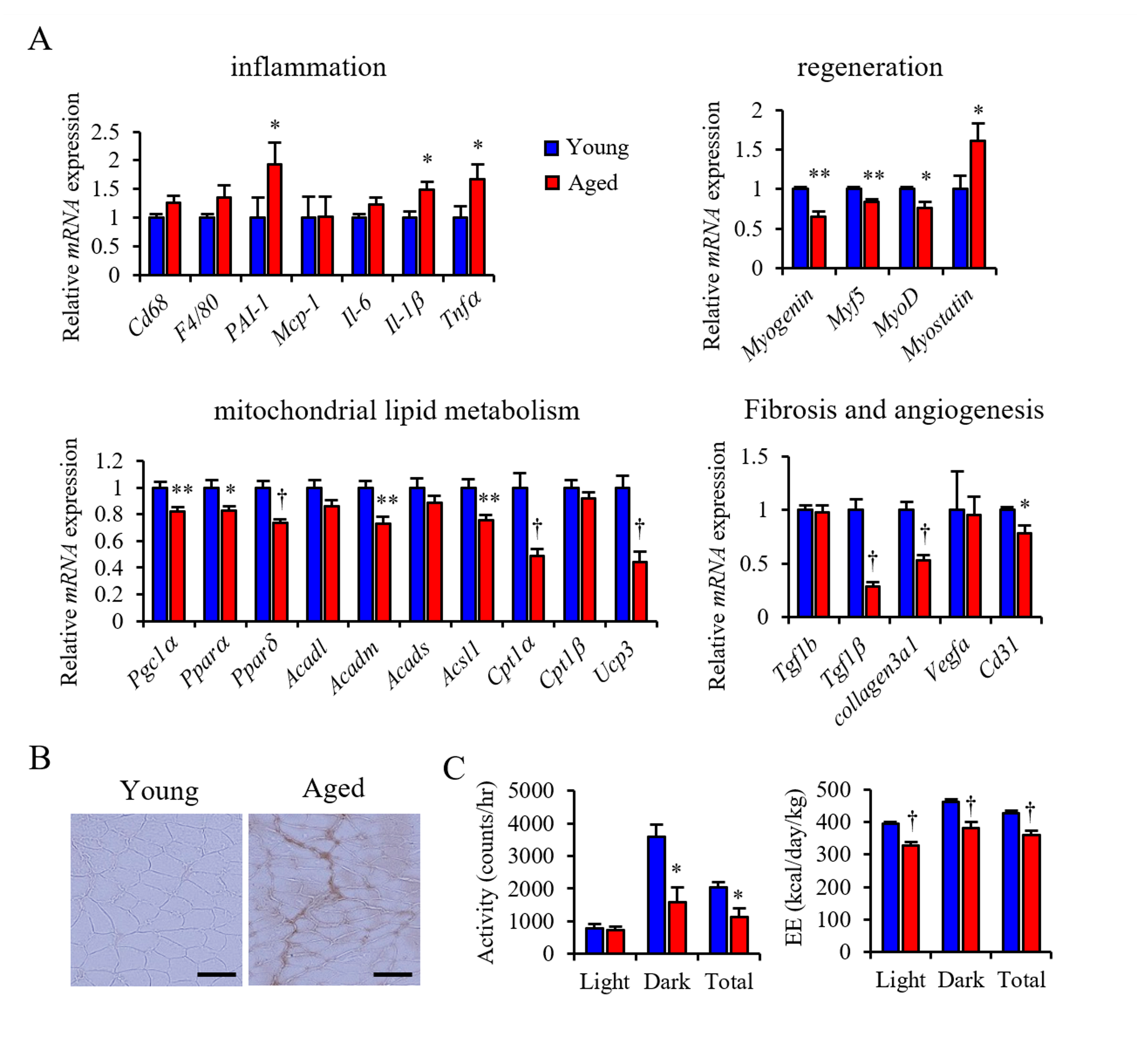

Supplement: S1 Fig — (A) Relative levels of transcripts marking inflammation (Cd68, F4/80, PAI-1, Mcp-1, Il-6, Il-1β and Tnfα), muscle cells differentiation and regeneration (Myogenin, Myf5, MyoD and Myostatin), mitochondrial lipid metabolism (Pgc1α, Pparα, Pparδ, Acadl, Acadm, Acads, Acsl1, Cpt1α, Cpt1β and Ucp3) and angiogenesis (Tgf1β, collagen1, collagen3a1, Vegfα and Cd31) in gastrocnemius of young and aged mice. Transcript levels were normalized to 18s mRNA; values in young mice were set to 1. (B) Representative images of 8-OHdG immunostaining to detect oxidative stress in gastrocnemius of young and aged mice. (C) Activity (left) and energy expenditure (EE) (right) in young and aged mice, as measured by indirect calorimetry. Young mice were 3-6-months-old and aged mice were 18-22-months-old. (Scale bar: 100μm in B). (n = 6–8 per group in A, C). All data are presented as means±S.E. Statistical significance was determined by Student’s t-test. *, p<0.05; **, p<0.01; †, p<0.001. (TIF) [file pone.0221366.s001.tif]

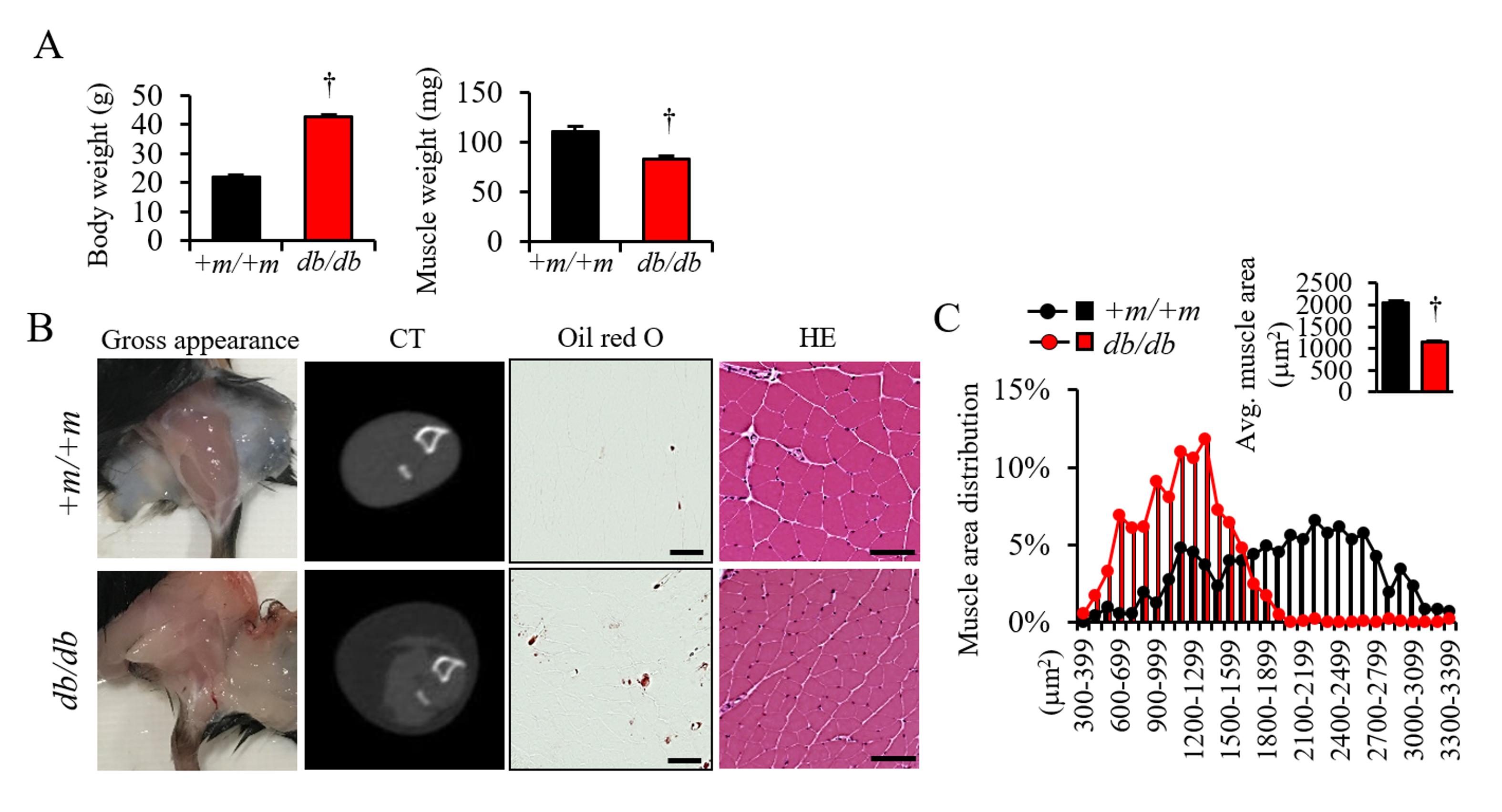

Supplement: S2 Fig — (A) Body weight (BW) and muscle weight (MW) of +m/+m and db/db mice (n = 6 per group). (B) Gross appearance of lower limbs and representative images of CT, Oil Red O staining and HE staining from +m/+m and db/db mice. Scale bar, 100μm (C) Quantification of muscle CSA in +m/+m and db/db mice (n = 1200–1500 per group). Data are presented as means±S.E. Statistical significance was determined by Student’s t-test. †, p<0.001. (TIF) [file pone.0221366.s002.tif]

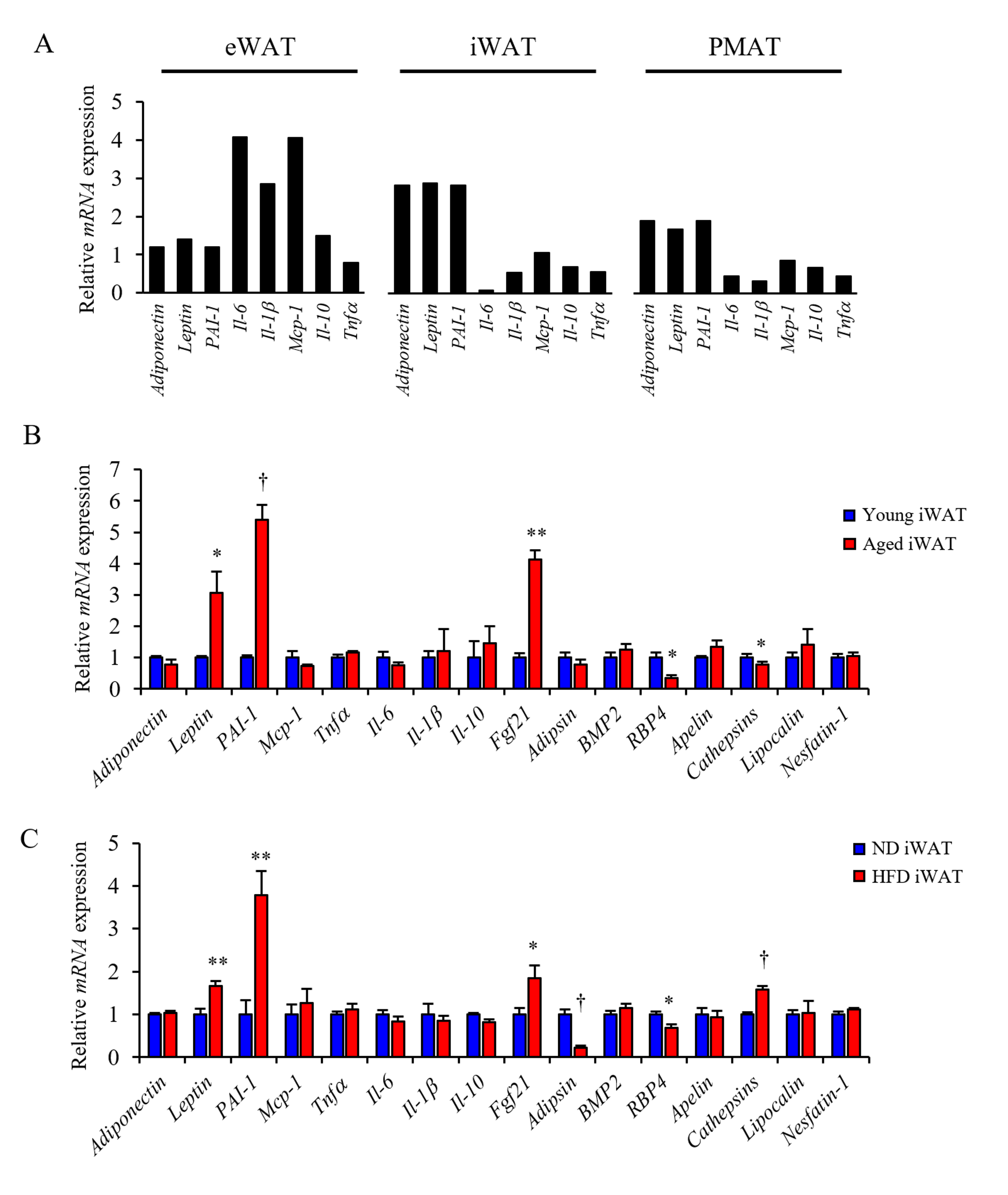

Supplement: S3 Fig — (A) Relative expression of transcripts encoding adipokines (Adiponectin and Leptin) and pro-inflammatory cytokines (PAI-1, Il-6, Il-1β, MCP-1, Il-10 and Tnfα) in eWAT, iWAT and PMAT from WT mice fed a HFD for 12 weeks (n = 2 per group). (B and C) Relative expression of genes associated with adipocytes or encoding pro-inflammatory cytokines in iWAT from young and aged mice (B), or from 12-week ND and HFD mice (C) (n = 6 per group). Transcript levels were normalized to 18s mRNA. Values in young iWAT or ND iWAT were set to 1. Data are presented as means±S.E. Statistical significance was determined by Student’s t-test. *, p<0.05; **, p<0.01; †, p<0.001. (TIF) [file pone.0221366.s003.tif]

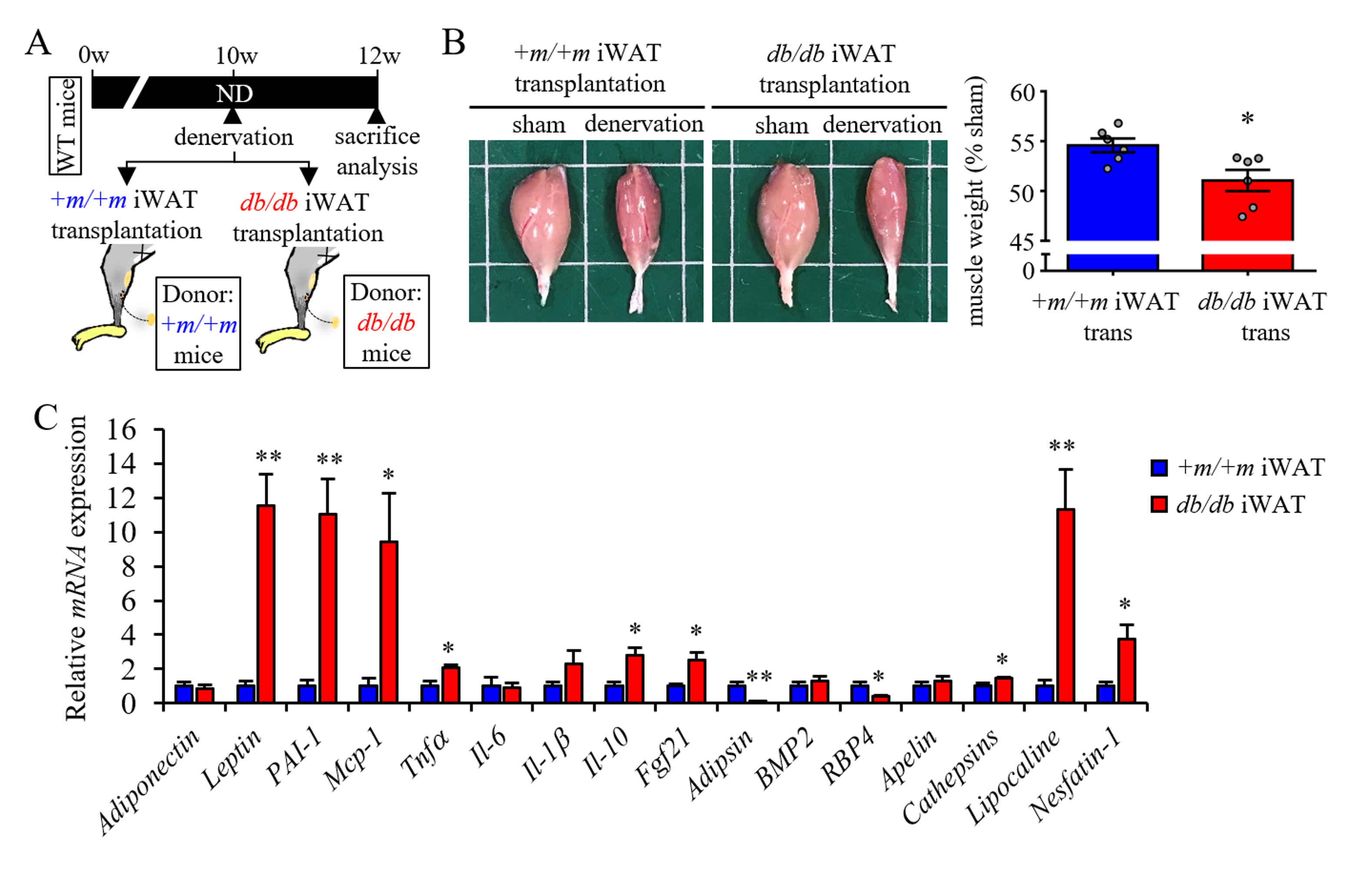

Supplement: S4 Fig — (A) Schematic illustrating the sciatic denervation model with iWAT transplantation from +m/+m or db/db mice. (B) Representative samples of gastrocnemius (left) plus quantification of tissue weight relative to sham (indicated as a %) in mice transplanted with iWAT from +m/+m or db/db mice after 2 weeks of denervation or sham operation. (C) Relative levels of mRNAs associated with adipocytes or encoding pro-inflammatory cytokines in iWAT of +m/+m or db/db mice (n = 6 per group). Transcript levels were normalized to 18s mRNA. Values in +m/+m mice were set to 1. All data are presented as means±S.E. Statistical significance was determined by Student’s t-test. *, p<0.05; **, p<0.01. (TIF) [file pone.0221366.s004.tif]

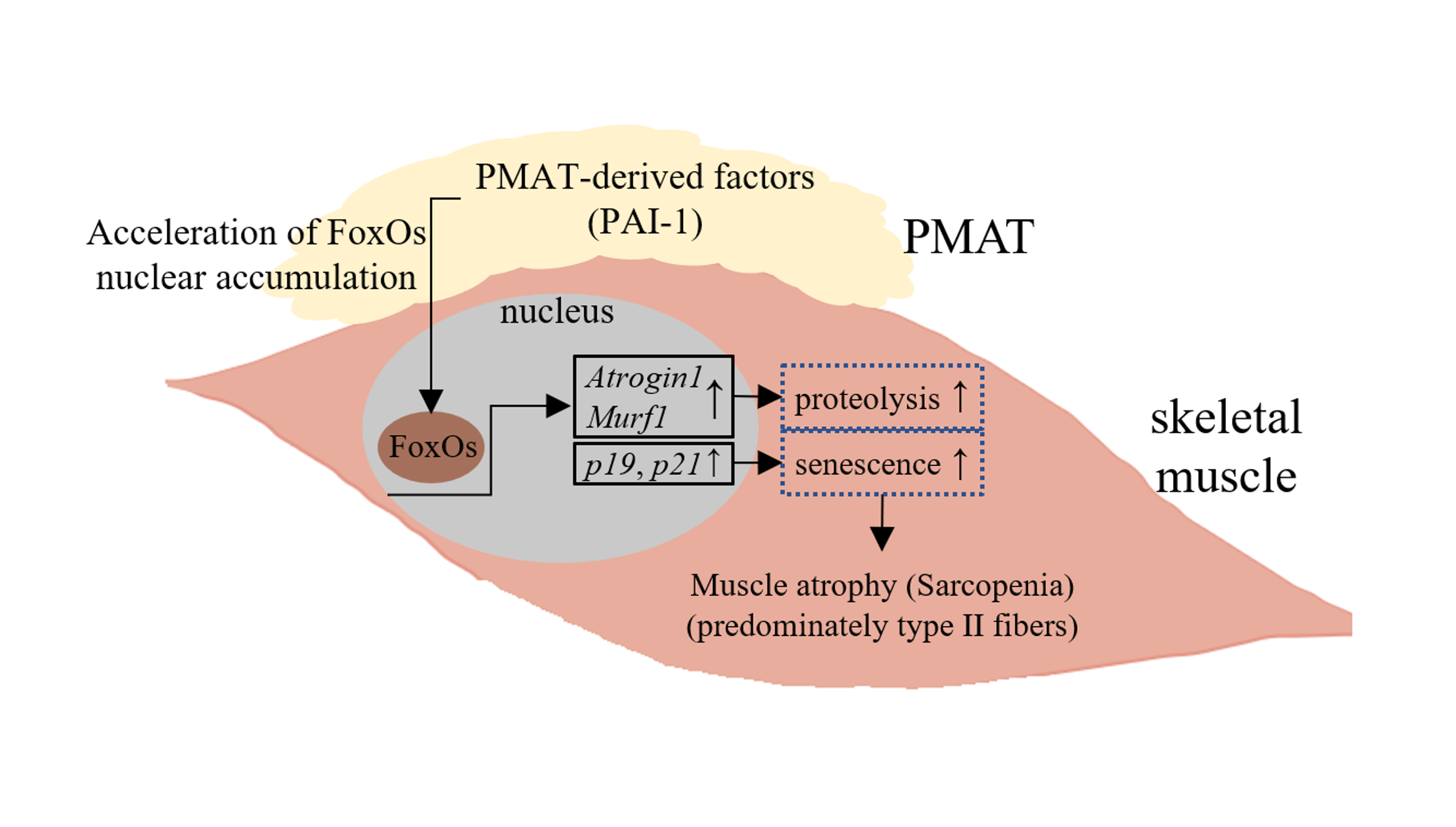

Supplement: S5 Fig — In aging or obesity, PMAT (in particular, PMAT secreting PAI-1) promotes nuclear translocation of FoxO transcription factors in skeletal muscle, accelerating skeletal muscle cell senescence and proteolysis and leading to atrophy (sarcopenia). (TIF) [file pone.0221366.s005.tif]
