# Supplementary material for: Aging- and obesity-related peri-muscular adipose tissue accelerates muscle atrophy
Source: PLoS One. 2019 Aug 23;14(8):e0221366. doi: 10.1371/journal.pone.0221366 (PMC6707561; doi:10.1371/journal.pone.0221366)
Supplement: S1 Table — (DOCX) [file pone.0221366.s006.docx]

**S1 Table**

Sequences of mouse primers used for quantitative RT-PCR

| Gene | Forward (5’-3’) | Reverse (5’-3’) |
| --- | --- | --- |
| *Rps18* | TTCTGGCCAACGGTCTAGACAAC | CCAGTGGTCTTGGTGTGCTGA |
| *p16* | CGAACTCTTTCGGTCGTACCC | CGAATCTGCACCGTAGTTGAGC |
| *p19* | GTTCTTGGTCACTGTGAGGATTCAG | CCATCATCATCACCTGGTCCAG |
| *p21* | TCAGAGCCACAGGCACCAT | TCCACGGGACCGAAGAGA |
| *p57* | TCTCTCGGGGATTCCAGGAC | CTTGCGCTTGGCGAAGAA |
| *Il-1β* | TCCAGGATGAGGACATGAGCAC | GAACGTCACACACCAGCAGGTTA |
| *Il-6* | CCACTTCACAAGTCGGAGGCTTA | GCAAGTGCATCATCGTTGTTCATAC |
| *Tnfα* | AAGCCTGTAGCCCACGTCGTA | GGCACCACTAGTTGGTTGTCTTTG |
| *Mcp-1* | GCATCCACGTGTTGGCTCA | CTCCAGCCTACTCATTGGGATCA |
| *F4/80* | GAGATTGTGGAAGCATCCGAGAC | GATGACTGTACCCACATGGCTGA |
| *PAI-1* | TTCCAAGGCATCCAGAAGCAG | CCGGAAATGACACATTGAAGTGAG |
| *Cd31* | CCGAAGCAGCACTCTTGCAG | CTGCAACTATTAAGGTGGCGATGA |
| *Cd68* | CATCAGAGCCCGAGTACAGTCTACC | AATTCTGCGCCATGAATGTCC |
| *Tgf1β* | TACGGCAGTGGCTGAACCAA | CGGTTCATGTCATGGATGGTG |
| *Vegfa* | GATGAAAGGCTTCAGTGTGGTC | GGGTTTGTCGTGTTTCTGGA |
| *Myostatin* | ATGGCCATGATCTTGC | GAGTGCTCATCGCAGT |
| *Myf5* | ACAGCAGCTTTGACAGCATC | GCTCTGTAGACGTGATCCGA |
| *MyoD* | ACGACACCGCCTACTACAGTG | TGGAGATGCGCTCCACTATG |
| *Myogenin* | AGTGAATGCAACTCCCACAG | GCTGTCCACGATGGACGTAAG |
| *Myh7* | CGGACCTTGGAAGACCAGAT | GACAGCTCCCCATTCTCTGT |
| *Myh2* | TGGAGGGTGAGGTAGAGAGTG | TTGGATAGATTTGTGTTGGATTG |
| *Myh1* | AGAAGCTCCTGGGATCCATT | CTCTCGCCAAGTACCCTCTG |
| *Myh4* | ACAGACTAAAGTGAAAGCC | CTCTCAACAGAAAGATGGAT |
| *Atrogin1* | ATGAGCTGCCCTATCTCAAGT | GTCCCGGTGTGTGTTCACAG |
| *Murf1* | CGGAAAGGTCTGCATCACAA | CCTTCATCAGGGCCTCAGTC |
| *Adiponectin* | GTCAGTGGATCTGACGACACCAA | ATGCCTGCCATCCAACCTG |
| *Leptin* | CAGGATCAATGACATTTCACACAC | CTGGTCCATCTTGGACAAACTC |
| *IL-10* | GACCAGCTGGACAACATACTGCTAA | GATAAGGCTTGGCAACCCAAGTAA |
| *Pgc1α* | CCGTAAATCTGCGGGATGATG | CAGTTTCGTTCGACCTGCGTAA |
| *Pparα* | ACGCTCCCGACCCATCTTTAG | TCCATAAATCGGCACCAGGAA |
| *Pparδ* | CAGATGACCCTTGTGCTGCCTA | TCTGACCCTGGGACCTAAGTGTG |
| *Acadl* | GGACTCCGGTTCTGCTTCCA | TGCAATCGGGTACTCCCACA |
| *Acadm* | CGAGTATGTTATCAACGGCCAGAA | GCGGGTACTTTAGGATCTGGGTTAG |
| *Acads* | AAGTTTGGATCCGCACAGCAG | CAAGCTTTGGTGCCGTTGAG |
| *Acsl1* | TTTGCCTGCAGCGAGTGTG | GCCCTCGACTATCCCTATGGTAAGA |
| *Cpt1α* | GCCATGATGGACCCCACAAC | CCAGATACTTGGACACCACATAGAGG |
| *Cpt1β* | GAGACAGGACACTGTGTGGGTGA | AGTGCCTTGGCTACTTGGTACGAG |
| *Ucp3* | GTGGTAAGCCATGCACACCTG | CCTGCTGCTTTGAACTGATGGA |
| *Fgf21* | AGATGGAGCTCTCTATGGATCG | GGGCTTCAGACTGGTACACAT |
| *Adipsin* | ACCTGACAGCCTTGAGGACGAC | GGGTTCCACTTCTTTGTCCTCG |
| *BMP2* | AACACCGTGCGCAGCTTCCATC | CGGAAGATCTGGAGTTCTGCAG |
| *RBP4* | TGTAGCCTCCTTTCTCCAGCGA | ACAGGTGCCATCCAGATTCTGC |
| *Apelin* | AGGCATAGCGTCCTCACCTCTT | GGTGCAGAAACGACAAAGACGG |
| *Cathepsins* | CATGACAAGCCTTCCTTCCAC | TTGTTCCCGTGCATCAAAG |
| *Lipocalin2* | ATGTCACCTCCATCCTGGTCAG | GCCACTTGCACATTGTAGCTCTG |
| *Nesfatin-1* | AGACCGATTGGTGACTCTGGAG | CCTCGGTGAATAACTGTTGCTGG |
| *Collagen1* | GAGCGGAGAGTACTGGATCGA | CTGACCTGTCTCCATGTTGCA |
| *Collagen3a1* | CAACCAGTGCAAGTGACCAA | GCACCATTGAGACATTTTGAAG |
